# Decreased electrophysiological activity represents the conscious state of emptiness in meditation

**DOI:** 10.3389/fpsyg.2014.00099

**Published:** 2014-02-17

**Authors:** Thilo Hinterberger, Stephanie Schmidt, Tsutomu Kamei, Harald Walach

**Affiliations:** ^1^Department of Psychosomatic Medicine, Research Section of Applied Consciousness Sciences, University Medical Center RegensburgRegensburg, Germany; ^2^Center for Industry, University and Government Cooperation, Nagasaki UniversityNagasaki, Japan; ^3^Institute for Transcultural Health Studies, Europa University ViadrinaFrankfurt (Oder), Germany

**Keywords:** EEG, meditation, consciousness, thoughtless emptiness, decreased neural processing

## Abstract

Many neuroscientific theories explain consciousness with higher order information processing corresponding to an activation of specific brain areas and processes. In contrast, most forms of meditation ask for a down-regulation of certain mental processing activities while remaining fully conscious. To identify the physiological properties of conscious states with decreased mental and cognitive processing, the electrical brain activity (64 channels of EEG) of 50 participants of various meditation proficiencies was measured during distinct and idiosyncratic meditative tasks. The tasks comprised a wakeful “thoughtless emptiness (TE),” a “focused attention,” and an “open monitoring” task asking for mindful presence in the moment and in the environment without attachment to distracting thoughts. Our analysis mainly focused on 30 highly experienced meditators with at least 5 years and 1000 h of meditation experience. Spectral EEG power comparisons of the TE state with the resting state or other forms of meditation showed decreased activities in specific frequency bands. In contrast to a focused attention task the TE task showed significant central and parietal gamma decreases (*p* < 0.05). Compared to open monitoring TE expressed decreased alpha and beta amplitudes, mainly in parietal areas (*p* < 0.01). TE presented significantly less delta (*p* < 0.001) and theta (*p* < 0.05) waves than a wakeful closed eyes resting condition. A group of participants with none or little meditation practice did not present those differences significantly. Our findings indicate that a conscious state of TE reached by experienced meditators is characterized by reduced high-frequency brain processing with simultaneous reduction of the low frequencies. This suggests that such a state of meditative conscious awareness might be different from higher cognitive and mentally focused states but also from states of sleep and drowsiness.

## Introduction

Meditation practices include a variety of different mental states or states of consciousness which are expressed through diverse EEG (electroencephalography) patterns, largely influenced by individual situational factors, observed meditation style and previous meditation experience. To enhance accuracy and comparability of meditation studies with respect to various meditative practices it is useful to discriminate between mental properties instead of meditation method or tradition. For example, Lutz et al. distinguished between two modes of meditation, the focused attention, and open monitoring (Lutz et al., 2008). They later described the practice of non-referential compassion as related to open monitoring but different with regard to the mental content, possibly representing a third category (Lutz et al., 2007).

All those concepts regard meditation as an engaged process requiring specific mental processes to be highly active. However, a consequent practice of meditation also asks for a quiet and effortless but fully conscious state which goes beyond the mentioned meditation types. Taking a look at common instructions for entering meditation we find that

- the mindfulness aspect of non-judgmental awareness asks for refraining from specific mental and conceptual elaboration of perceptions while staying fully conscious
- the mindfulness aspect of being present in the moment asks for refraining from planning and the mental construction of time
- an advanced state of thoughtlessness, sometimes experienced as “pure being” or a state close to so called “non-duality” is characterized by being conscious however without apparent categorical thinking or episodic memories.

These aspects of meditation ask for the ability to down-regulate certain mental processing activities. This would constitute a unique mode of meditation which is expected to present different neuronal patterns compared to focused attention or open monitoring (including compassion). It would suggest a reduction of brain processing functions, an assumption born out of an imaging study of Zen meditators that documented less default-mode-network (DMN) activity in meditators compared to controls and a quicker return to a lower baseline-activity after a task dependent activation (Pagnoni et al., 2008). Travis and Shear classified meditation techniques into three categories namely focused attention, open monitoring, and automatic self-transcending (Travis and Shear, 2010). They state: “Focus and monitoring experience are active mental processes, which keep the brain engaged in specific processing—individual activity keeps the mind from transcending.”

In the present study we focus on an aspect of meditation that connects to automatic self-transcending which we refer to as a state of “thoughtless emptiness” (TE) or “thoughtless void.” TE might be close to the conception of the Vedic form of “advaita” or what some call “non-duality.” It requires refraining from using thoughts, memories, emotions, associations, perceptions while consciousness is maintained. Thus, this state is supposed to express reduced mental processing as described above. We were interested in identifying the neurophysiological properties of such “pure” consciousness. Therefore, TE not only had to be distinguished from meditative states of consciousness associated with higher order processing but also from (a) idling brain states which are present in resting conditions and (b) those states that lead to a loss of consciousness (LOC) such as sleep onset.

Before presenting own results a short overview about the expected brain dynamics of various states measured by the EEG is given.

### The resting state network

Resting task conditions were described by the activation of resting state networks (RSN) which represent mental functions of memorization, planning, evaluation of potentially survival-salient information from the body and the world, and self-referential functions. One of the RSNs is called the default mode network (DMN). DMN activity is reduced during attention-demanding cognitive processing (Raichle et al., 2001). Jann et al. (2010) found 10 different RSNs one of which is the DMN. Its activity was associated with increased alpha1 activity in the central areas and partly frontal areas, increased alpha2 in the posterior occipital region, increased beta1 over parietal electrodes, decreased delta at fronto-central and decreased theta in parieto-occipital areas.

### Sleepiness and sleep

Feelings of sleepiness during prolonged wakefulness are related to increased theta power, mainly frontally, and a decrease in alpha power (Strijkstra et al., 2003). A shift from dominant alpha to theta activity (3–7 Hz) indicates the transition from wakefulness to sleep stage 1. Stage 2 shows a distinct EEG pattern consisting of so-called sleep spindles (12–14 Hz) and K-complexes. During stage 3, or slow-wave sleep, delta activity (0.5–2 Hz) increases (Iber et al., 2007). Whereas these first three sleep stages are typically classified as unconscious non-REM (rapid eye movement) sleep, REM sleep is conscious and characterized by a predominance of theta activity (Rechtschaffen and Kales, 1968). Fronto-medial theta is thought to be related to the occurrence of dream images (Inanaga, 1998). Ogilvie et al. found significant increases over all standard EEG frequencies at the time of sleep onset (Ogilvie et al., 1991; Klimesch, 1999).

### Various meditation modes

Mindfulness meditation emphasizes enhanced awareness and mindfulness, thus it is often thought to be a state of heightened wakefulness and attentiveness. One facet of mindfulness is the state of being present; another is the state of enhanced acceptance (Kohls et al., 2009). Mindfulness thus falls into the category of open monitoring and is related to our experimental condition of presence. Activations of brain regions involved in attentional control (e.g., prefrontal cortex), conflict resolution (e.g., dorsal anterior cingulate cortex), and emotional processing (e.g., medial/orbitofrontal cortices) support these aspects of meditation (Hölzel et al., 2007; Baron Short et al., 2010). Attention correlates positively with frontal midline theta band power (Gevins et al., 1997; Aftanas and Golosheikine, 2001; Başar et al., 2001). In contrast to sleep related theta activity, the task-related increases occur within a small frequency range (Klimesch, 1999). Klimesch suggested that broad band theta synchronization as observed during sleep lowers or blocks the ability of information encoding, whereas narrow band synchronization is closely linked to the encoding of information.

Lehmann and colleagues observed that a highly experienced Tibetan lama exhibited strong 40 Hz gamma oscillations arising from different oscillators depending on the type of meditation practiced, with the “dissolution of self” meditation activating right prefrontal anterior centers (Lehmann et al., 2001). Increased gamma power was also reported for meditators during focused concentration on an object (Lutz et al., 2003) as well as unconditional loving kindness and compassion compared to the resting state (Lutz et al., 2004). Generally, gamma oscillations around 40 Hz have been associated with cognitive processing and the temporal binding of perceptual stimuli (Llinás and Ribary, 1993; Joliot et al., 1994; Lutzenberger et al., 1995; Elliott, 1998; Kaiser and Lutzenberger, 2005). For a review of various studies related to meditation also see (Cahn and Polich, 2006).

Travis and Shear's analysis of EEG correlates of their three proposed meditation states suggests that focused attention was characterized by beta and gamma activity, open monitoring by theta activity and automatic self-transcending by lower alpha activity (Travis and Shear, 2010).

The aim of our study was to characterize the electrophysiological correlates related to various meditative states of consciousness. For this purpose, we have asked meditators from different traditions and with varying proficiency to meditate while measuring 64 channels of EEG and to enter a variety of intended states: a state of *resting* wakefulness, an open-monitoring-like state of highest possible presence (*presence/monitoring*), a state of *focused attention*, the state of *TE*, and finally an attunement to a first visualized and then generally perceived *spatial connectedness*. The basic question in this study was to identify those brain processes which remain active even in a state of *TE* with reduced mental processing and are required for staying awake without falling asleep. Besides contrasting the EEG spectra of the measured states against each other we additionally compared the results from participants with high and low meditation proficiency.

## Materials and methods

### Participants

#### Sociodemography

Altogether, we collected data from 50 participants aged from 22 to 68 years (mean age 45 years, 17 females/33 males). All participants were subjected to the same experimental procedure described above. The meditating participants were eligible for inclusion in the study if they had been carrying out a meditative spiritual practice on a regular basis for at least 2 years. To extend the range of meditation experience to the lower end, eight age-matched participants without meditation experience were included.

The meditator participants were associated with different kinds of spiritual traditions such as Zen-Buddhism (11), Qi-Gong (4), Tibetan Buddhism (4), Sahaja Yoga (8), Western contemplative methods (7), spiritists or mediumistic practice (5), or were spiritual healers or shamans (3). Six participants were Zen Buddhist monks in Japan. Most of the Buddhist and Qi-Gong practitioners were Japanese or Chinese and were measured in Japan. Based on these techniques, the subjects developed their individual “idiosyncratic” meditation style. All participants participated voluntarily and received no remuneration. The study was approved by the ethics committee of the University of Northampton/UK and the ethics committee of the University Medical Center Freiburg i.Br./Germany.

#### Meditation experience

The total meditation experience was the criterion for grouping of participants. It was calculated by multiplying the years of experience with their weekly practice sessions and the time per session. The results for the 50 participants varied between 0 and 21,185 h. Meditation experience averaged 3357 h and 14 years of meditation practice. While 8/50 participants had no meditation experience. 4/50 meditating participants had less than 50 h of experience, 7/50 had between 100 and 1000 h, 18/50 between 1000 and 5000 h, 6 between 5000 and 10,000 h and 7 participants had more than 10,000 h of meditation experience in their life.

#### Grouping

For the analysis of meditation-specific states we intended to use only experienced meditators. Thus, we included all participants with at least 5 years practice or more than 1000 h of total meditation time, which applied to 30 subjects (11 females/ 19 males, mean age 47 years). On average, participants in this group had meditated for 20 years and 6498 h.

The influence of meditation experience on the findings was researched by a between-group comparison. Based on total meditation practice hours, the group of all 50 available participants was divided into three subgroups of equal size. For the between-group analyses, the group of 17 most experienced meditators (MEM) was compared to the group of 17 least experienced meditators (LEM), the intermediate group was omitted. The 17 LEM (4 females/13 males, mean age 42 years) had less than 500 h of meditation practice (mean practice of 111 h and 3.4 years); the 17 MEM (5 females/ 12 males, mean age 48 years) had at least 3800 h of meditation practice (mean practice of 9716 h and 27 years).

### Experimental procedure

All participants were informed about the aims of the study and gave their consent before their participation. A brief initial questionnaire asking questions about meditative experience in terms of duration and type of practice was administered. In addition, they were asked to describe the posture and method of their meditative practice as precisely as possible. Three recording sessions were carried out with each participant:

1. The recording started with an initial 15 min baseline session in which participants should *rest* but not meditate while sitting in their usual meditation posture for 5 min with eyes opened, 5 min with eyes closed, and spend 5 min silently *reading* a text from a book or a computer screen.
2. After a short break, a meditation session of 20–30 min duration was carried out in which they were asked to meditate in their own usual way, from now on referred to as “*idiosyncratic meditation*.” The idiosyncratic meditation is the only state in which subjects were not instructed to aim for a specific mental state, but could choose their own specification. The subjects' reports of commonly used meditation techniques include mindfulness/presence/awareness (10), thoughtlessness/emptiness (10), visualization/Chakra (10), connectedness/unity (4), Kundalini Yoga (6). Their meditation practice also included mediumship, Mantras, concentration, connecting with god or an energy source. Participants without prior meditation experience received a short introduction into meditation. They were instructed to sit as relaxed as possible in an upright position while focusing on their breath and letting their thoughts come and go with a non-judgmental attitude. Special events, feelings, or states of consciousness occurring during the meditation could be signaled by the participants by pressing a button.
3. Subsequently, an additional guided short meditation session of 10 min duration was performed, comprising another four tasks, each lasting 2 min. All subjects had their eyes closed. The denotations are listed together with the corresponding instructions as follows:
  - *Presence/Monitoring*: “Try to be in a state of high presence at the place you are in this room at each moment of time.”
  - *Thoughtless emptiness (TE)*: “Try to maintain the state of emptiness from all thought as well as possible.”
  - *Focused attention*: “Direct your attention on a spot in the middle of the forehead above your eyes. This spot is sometimes called ‘the 3rd eye’ if you are familiar with this term.”
  - *Spatial connectedness*: “Sit in an upright position. First visualize and then just perceive an interconnecting ‘energy stream’ going through the body axis, and its projection down to earth and up to the sky.”

Instructions were spoken by the same experimenter for all participants to initiate the task during the recording. Before the recording the instructions were explained by the experimenter and participant had the chance to ask questions. All participants reported that they could follow or were familiar with the meditative tasks requested in this study.

By offering these different tasks we intended to operationalize four supposedly different mental states operative in meditation, also requiring concentration and mindfulness, which might be related to the executive and the monitoring function of attention-networks (Austin, 2014). Events, feelings, emotions, thoughts and properties of the session were summarized in a written report after the sessions. For the analysis of instructed meditation tasks it was important to verify that the meditators were able to reach and maintain the requested mental states. This was accomplished by asking them after completion how well they were able to fulfill the task. All meditators reported successful implementation. An entire session lasted about 2.5–3 h.

### Experimental setup

All participants were measured with the same equipment. During all sessions, physiological data were recorded with a 72 channels QuickAmp amplifier system (BrainProducts GmbH, Munich, Germany). EEG was measured using a 64 channels ANT electrode cap with active shielding and Ag/AgCl electrodes which were arranged according to the international 10/10 system. The system was grounded at the participants' shoulder. Data were recorded at a sampling rate of 250 Hz and 22 bit resolution with a common average reference and filtered in a range from DC to 70 Hz. For correction of eye movement and blink artifacts, the vertical electrooculogram (EOG) was measured by placing two electrodes above and below one eye. Respiration was measured with a respiration belt and skin conductance at the second and third finger of the non-dominant hand. Additionally, for measuring heart rate variability, the electrocardiogram (ECG) was assessed with two more electrodes. The experimenter was monitoring the raw data during the recording. Due to meditators residing in different countries, the measurements were carried out at various locations, predominantly in rooms normally used for meditation or in the participants' homes.

### Processing of physiological data

#### Preprocessing

The whole data analysis was done using Matlab version 7.3. After detrending the DC recorded EEG data sets all EEG channels were corrected for eye movements using a linear correction algorithm as described in Hinterberger et al. (2003). This algorithm detects eye blinks and movement events and uses those periods for determining a correction factor for each channel. The EOG was multiplied with this factor and then subtracted from the EEG according to Gratton et al. (1983). This algorithm was tested to work sufficiently well in normal non-moving EEG and can also be applied in real-time online analysis (Hinterberger et al., 2003). The time-series of the raw data and also spectral data was visually inspected for continuous artifacts by means of the visualization scheme used in the state monitoring approach (Hinterberger, 2014). Periods with non-local artifacts involving many electrodes were eliminated. Local artifacts such as bad electrodes were eliminated by replacing the signal with an interpolation of the surrounding electrodes. This was the case in two participants. The remaining possible short-term high-amplitude artifacts were handled in the temporal averaging procedure, e.g., by using the median instead of the mean as described below.

#### Power spectral density (PSD)

A power spectrum time series was calculated using the Fast Fourier Transform (FFT). FFT was applied to the windowed EEG time series which was convolved using a Nutall window and shifted in steps of 0.5 s. A window size of 2 s was chosen for calculation of the FFT frequency coefficients up to 10 Hz while all higher frequencies were calculated using a 1 s window. The Fourier amplitudes of the 1 s windows had to be multiplied by the square root of 2 to make them comparable with the 2 s windowed frequency coefficients. To limit the influence of high amplitude artifacts the spectral amplitudes were limited to five standard deviations.

It has been shown that the selection of standard frequency bands does not result in an optimal independency of the bands (Doppelmayr et al., 1998). Since the rhythms slow down with age, it would make sense to define the frequency band limits individually for each subject (Köpruner et al., 1984). Therefore, in our approach an algorithm was developed that allowed for determining the individual alpha peak frequency (IAF) using the resting EEG data stream stemming from the eyes closed condition because alpha is most dominant in an *eyes closed resting* state. For calculation of the IAF the algorithm carried out the following steps:

1. Searching for the 1 min epoch in the eyes closed data with the highest standard alpha band activity (8–12 Hz) in parietal-occipital areas. This epoch served as analysis epoch for the IAF.
2. Extracting this epoch from unfiltered pre-processed raw data (corrected for EOG artifacts).
3. Calculating a high-resolution FFT over the 1 min epoch in the full frequency range from 0 to 70 Hz.
4. Averaging of all spectra from parietal and occipital regions.
5. Determining the individual expected age-dependent peak frequency as IAFexp = 10.89 − (age − 20)/50 ∗ 2.65). According to Doppelmayr et al. (1998) the actual IAF can vary, influencing also the range of the theta band and consequently the upper limit of the delta band. Thus, the IAF was determined using the high-resolution spectra and searching for a peak in the range of IAFexp ± 2 Hz.

Using the IAF as an anchor, the frequency bands were determined as follows:

Delta: 1.5 Hz to 0.6 ∗ IAF
Theta: 0.6–0.8 ∗ IAF
Alpha: 0.8–1.2 ∗ IAF
Beta1: 12.5–16 Hz
Beta2: 16.5–24 Hz
Gamma1: 24.5–47 Hz
Gamma2: 54.0–70 Hz

To obtain a measure of the power spectral density (PSD) FFT values were squared and all FFT bins within a frequency band range were averaged. EEG PSD was calculated for each participant, recording session, electrode, and frequency band.

The subsequent statistical comparisons of EEG PSD measures using *t*-tests require the assumption of having normally distributed data. To ensure statistical validity, it was necessary to take a closer look at the distribution of the data sets. Therefore, a distribution analysis of the EEG measures, especially the PSD measures was done. As a result, the log- transformed Fourier amplitude values (as used for PSD) could be fitted with a normal distribution function in a wide range so that more than 99% of the values were within the normal distribution. Therefore, all PSD values were log-transformed for further statistics.

#### Temporal averaging procedure

**Figure 1:**
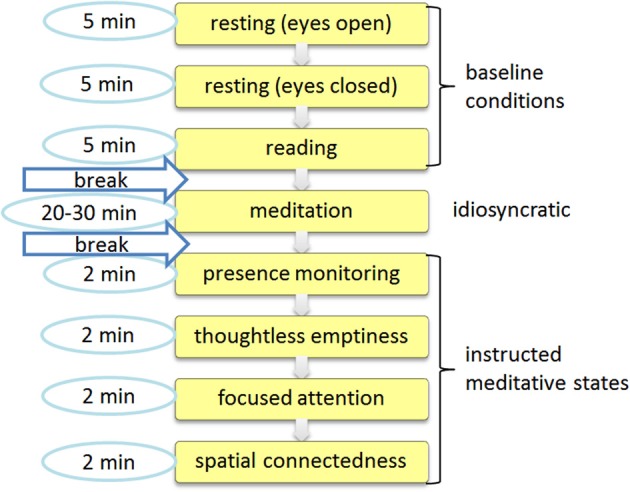
**Session protocol**. The session consisted of an initial baseline recording that was followed by an idiosyncratic meditation task and a subsequent session with four specific meditative tasks.

For further data analysis artifact-free epochs of the eight conditions (according to Figure 1) were selected and averaged. In order to be robust against rare but possible high amplitude artifacts the temporal median was used and the interquartile range served as a measure for the standard deviation which can be estimated by multiplication with 0.7413.

In order to compare the *idiosyncratic meditation* with normal *resting* state activity a further artificial reference condition was defined as either the eyes opened or eyes closed condition, or, when participants chose to have their eyes half open during the *idiosyncratic meditation*, the mean values between eyes opened and closed resting were used. This adjusted additional resting state is marked with an asterisk (resting^*^). Those 8 + 1 conditions are now called task conditions. They were available for each participant and can therefore be compared with each other.

#### Spatial data reduction

**Figure 2:**
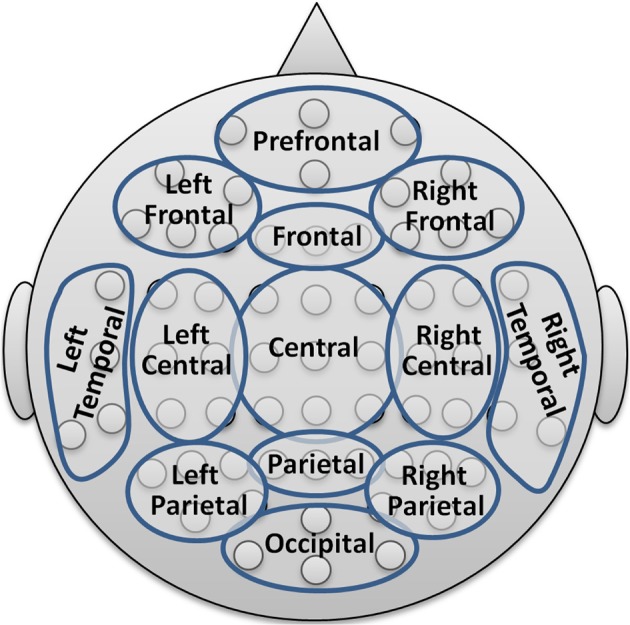
**Reduction scheme into 13 major topographic brain regions**.

To limit the number of coefficients in the statistical analysis and for providing a comprehensive visualization with spectral and topographical resolution, the 64 channels were merged into 13 topographic brain regions according to Figure 2.

The resulting PSD data set for each participant thus comprised the dimensionality of 8 + 1 conditions times 13 areas times 7 frequency bands. Further reduction levels were achieved by averaging over all electrodes resulting in the global band power (GBP) and further averaging the GBP measures over all bands leading to a global field power (GFP) measure.

#### Comparison between conditions

To limit the number of task comparisons to those relevant for answering our research questions, the following eight comparisons were selected:

1. Eyes opened resting vs. eyes closed resting.
2. Reading vs. eyes opened resting.
3. Idiosyncratic Meditation vs. resting^*^.
4. Presence/monitoring vs. resting (eyes closed).
5. TE vs. resting (eyes closed).
6. TE vs. presence/monitoring.
7. TE vs. focused attention.
8. TE vs. spatial connectedness.

The temporal medians of the power spectral estimates of the respective conditions are compared by calculation of effect sizes defined as standardized mean differences (Cohen's d)^1^. For calculation of the group effect, the effect sizes of all participants were submitted to a paired two-tailed *t*-test across participants for each location and frequency band.

#### False discovery rate adjustment

On the level of GBP there are seven frequency bands and eight comparisons resulting in 56 variables. On the level of spatially resolved data of 13 areas statistics provides 728 variables, meaning that 36 values should randomly reach significance on a 0.05 level. Correction for multiple comparisons is not trivial as such measures may be dependent on each other. Therefore, Bonferroni correction of significance levels would be far too conservative and wipe out most effects. The false discovery rate (FDR) gives the proportion of false discoveries among all discoveries. Based on the formulas by Benjamini and Hochberg (1995) and Yekutieli and Benjamini (1999), we applied FDR adjustment on all three dimensions, i.e., on the level of 7 frequency bands, 13 brain areas, and 8 task comparisons.

#### Summary of data sets

In summary, the presented statistical results comprised the following PSD differences:

- The GFP as an average over all electrodes and all frequencies of the 8 comparisons (*N* = 30 experienced meditators).
- The global band power (GBP) as the average PSD over all electrodes of 8 comparisons and 7 frequency bands (*N* = 30 experienced meditators).
- Spatially resolved PSD from 8 comparisons, 7 frequency bands, and 13 topographic brain regions (*N* = 30 experienced meditators).
- The GBP as the average PSD over all electrodes, providing one data matrix of 8 comparisons × 7 frequency bands that includes 17 LEM and on including 17 MEM.

### Research questions

On the basis of this data and with respect to the introduced issues, we formulated the following exploratory research questions:

(**Q1**) **Global PSD comparisons**. What are the global differences in PSD between all comparisons of interest? This will give a first impression of the findings on a high reduction level.
(**Q2**) **Detailed PSD comparisons**. What are the spectral and topographical characteristics of the EEG PSD differences when comparing
  - *resting conditions* with selected states?
  - *thoughtless emptiness (TE)* with other standardized conditions?
(**Q3**) **Idiosyncratic meditation compared**. How do the idiosyncratic meditation styles correspond to the standardized instructed conditions?
(**Q4**) **Impact of experience**. How does meditation experience influence PSD differences?
  - In *group comparisons*, the 17 least and 17 MEM were compared.
  - A *regression analysis* over all 50 participants could uncover the dependency of experience and state differences.

## Results

The first section presents the brain activities related to different mental states in the group of 30 experienced meditators. The second section illustrates the comparisons between the LEM and MEM.

### Global PSD comparisons (Q1)

First, a general description of the characteristics of all eight state comparisons is provided. On the highest reduction level, the GFP includes all frequencies between 1 and 70 Hz and an average over all electrodes. *t*-test comparisons between task conditions were calculated and displayed in Table 1. To correct for multiple testing in 8 different comparisons and 7 frequency bands, an FDR adjustment was applied to the resulting *p*-values. The corrected *p*-values are shown in Table 1 and *p*-values in the text refer to the corrected *p*-values.

**Table 1:** **State comparisons on the level of the global field power have been calculated**.

**State comparisons**	***t* (*n* = 30)**	***p*-value^+^**
Eyes open vs. eyes closed resting	−2.93^**^	0.010
Reading vs. eyes open	−1.45	0.219
Idiosyncratic meditation vs. resting^*^	−3.40^**^	0.006
Presence/monitoring vs. resting (eyes closed)	−0.37	0.844
TE vs. resting (eyes closed)	−3.14^**^	0.001
TE vs. presence/monitoring	−3.91^**^	0.004
TE vs. narrow focusing	0.11	0.844
TE vs. spatial connectedness	−0.33	0.844

Resting

* condition depends on eyes open or closed in idiosyncratic meditation,

** corrected p ≤ 0.01.

+ The p-values were corrected for multiple comparisons.

There was a significant overall EEG power decrease during the *idiosyncratic meditation* condition as compared to the *resting* state (*t* = −3.4, *p* < 0.01). GFP also decreased significantly during *TE*, compared to *resting* (*t* = −3.14, *p* < 0.01) and compared to *presence/monitoring* (*t* = −3.91, *p* < 0.01). In other words, the state of *presence/monitoring* has significantly greater overall EEG power than *TE*.

**Figure 3:**
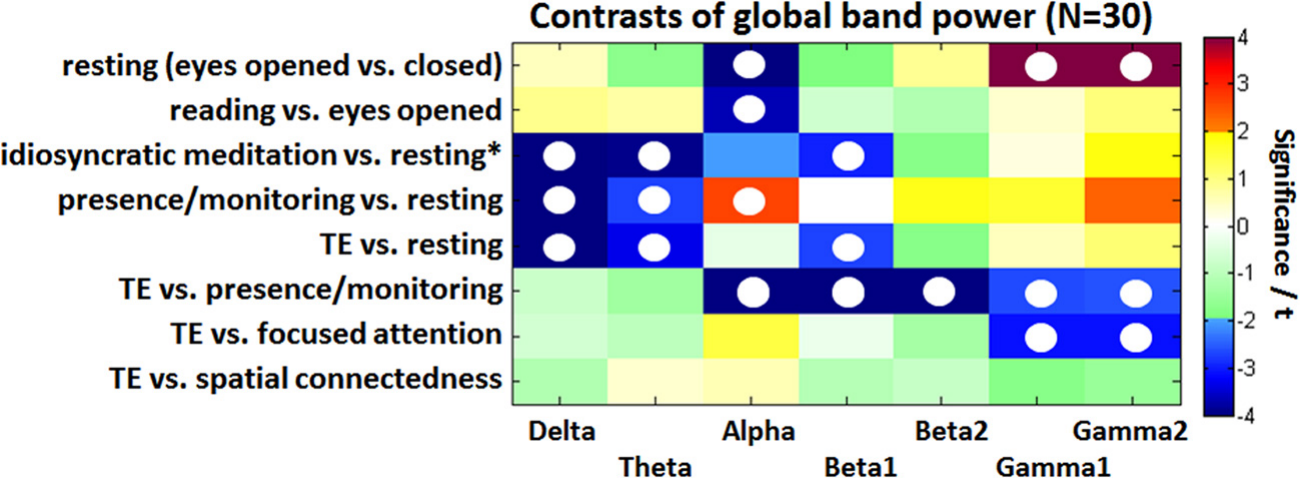
**Color-coded PSD differences shown as *t*-values resulting from the eight standard comparisons between psychophysiological states during the recording sessions for seven distinct frequency bands, averaged over 30 experienced meditators**. The colors are coding *t*-values. All orange-red fields are positively significant on a 5% level and all bluish values are negatively significant. White, yellow, and green fields are non-significant. Fields marked with a white circle were significant on the 0.05 level after FDR adjustment.

A spectrally resolved view on these results is illustrated in Figure 3 presenting the GBP. The alpha and gamma differences between *eyes open* and *eyes closed resting* are a highly significant but also trivial result. All three comparisons against the *resting* condition (*idiosyncratic meditation, presence/monitoring*, and *TE*) show that normal *resting* is associated with increased delta and theta waves compared to meditative states. The GBP during *idiosyncratic meditation* related to *resting* is very similar to the contrast between *TE* and *eyes closed resting*. While the beta1 band offers a reduction during *idiosyncratic meditation* (*t* = −2.8, *p* < 0.05) and *TE* (*t* = −2.7, *p* < 0.05), the state of *presence/monitoring* is associated with an increased alpha (*t* = 2.7, *p* < 0.05). The comparison of *TE* with each of four conditions shows quite characteristic differences: while the *resting* state expresses higher delta (*t* = 6.0, *p* < 0.001), theta (*t* = 3.3, *p* < 0.05), and beta1 (*t* = 2.7, *p* < 0.05) activations, the state of *presence/monitoring* strongly differs in the alpha (*p* < 0.01) and beta (*p* < 0.01) range and less in the gamma range (*p* < 0.05). A narrow, but not visual focus (*focused attention* condition) only affects gamma activity (*p* < 0.05). The *spatial connectedness* exercise is not significantly different from the state of *TE*.

### Detailed PSD comparisons (Q2)

#### Differences to resting states (Q2a)

**Figure 4:**
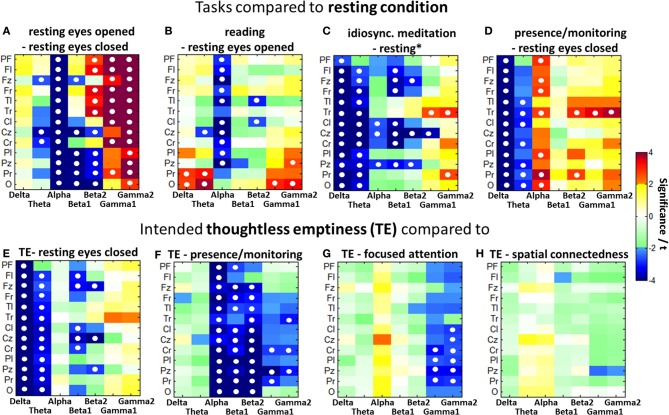
**Color-coded PSD differences shown as *t*-values resulting from eight selected task comparisons (A–H). (A)** *t*-test of 30 experienced participants was calculated from the effect sizes (Cohen's d) from each participant for each location and frequency band. Fields marked with a white circle reached significance on the 0.05 level after FDR adjustment over all three dimensions, i.e., brain regions, frequency bands, and task comparisons.

Besides the global alpha blocking in the *eyes open* vs. *eyes closed* condition *p* < 0.001), Figure 4A) shows a reduction in parietal-occipital PSD in the alpha (*p* < 0.001), beta1 (*p* < 0.01), and beta2 (*p* < 0.05) bands, while frontal and temporal beta2 power increased (*p* < 0.05). Large gamma increases with opened eyes predominantly show up in lateralized frontal (*p* < 0.001), central (*p* < 0.01), and temporal (*p* < 0.01) areas. The cognitive load induced through reading further decreased the alpha rhythms (*p* < 0.05) while parietal and occipital theta (*p* < 0.05) and occipital gamma (*p* < 0.05) increased significantly (Figure 4B).

Contrasting the *resting* condition with meditative states as shown in Figures 4C–E all those comparisons present almost globally lowered delta and theta band PSD. While the *idiosyncratic meditation* offers tendencies toward decreased alpha (central and parieto-central *p* < 0.05), the eyes closed *presence/monitoring* condition presents clearly increased alpha power (*p* < 0.05) predominantly on the right side. All meditative styles present more or less increased temporal gamma power.

#### Differences of thoughtless emptiness (TE) (Q2b)

Figures 4E–H show the comparisons between *TE* and the *resting* condition as well as the other three investigated meditative states. For all comparisons, the differences within one frequency band tend to occur globally over all brain areas in the same direction.

Compared to the *resting* state, *TE* is related to a significant decrease in the delta frequencies for all brain areas (*p* < 0.001) and in the theta frequencies for all but the prefrontal area (*p* < 0.05). Beta1 is decreased mainly in the frontal (*p* < 0.05) and central (*p* < 0.05) areas and beta2 in the frontal midline (*p* < 0.05), central midline (*p* < 0.001) and parietal midline (*p* < 0.05) regions. The state of *TE* has globally decreased delta and theta only in comparison to the *resting* state, but not compared to the other meditative states.

Compared to the state of *presence/monitoring*, *TE* shows significantly decreased activation in all brain areas for alpha (*p* < 0.01) and in most areas for beta1 (*p* < 0.01) frequencies, as well as in all but the prefrontal and left frontal areas for beta2 (*p* < 0.01) and the right temporal (*p* < 0.05), right central (*p* < 0.05) and mid-parietal (*p* < 0.05) areas for the gamma band. The *presence/monitoring* state is the only state to have higher alpha power than TE.

In comparison with the *focused attention* condition, *TE* shows significant central and parietal gamma decreases (*p* < 0.05) even after FDR adjustment.

The *spatial connectedness* has the greatest similarity in brain activity to the state of *TE*. No significant differences are expressed after FDR adjustment. Without this correction *spatial connectedness* offers a higher gamma activity in the central parietal area compared to *TE*.

### Thoughtless emptiness and idiosyncratic meditation (Q3)

When assessing individual meditation styles which built the base for the *idiosyncratic meditation*, 10/30 mentioned a kind of TE as their primary aim. Another 10/30 used mindfulness techniques, 4/10 visualizations, and again 10/30 reported of the experience of connectedness or unity. As most meditators reported several contents within their meditation session and the experiences could hardly be addressed to only one specific state we decided to treat all *idiosyncratic meditation* sessions of all 30 meditators as an undefined and pluralistic condition.

A comparison of the power spectral patterns of the 20–30 min *idiosyncratic meditation* and the intended *TE*, both contrasted against a resting task as visible in Figures 4C,E, presents large similarities. *TE* shares many features with the *idiosyncratic meditation*. Both conditions offer globally decreased delta and theta band activities as well as frontal and central beta-band reductions. In both conditions, also right temporal gamma increases significantly. The central alpha desynchronization during *idiosyncratic meditation* is not visible in the *TE* condition.

### Proficiency dependent PSD differences (Q4)

#### Analysis of IAF

Aftanas et al. reported a lower IAF for long-term meditators (3–7 years) relative to short-term meditators (Aftanas and Golosheikin, 2003). Our data do not support these findings. The group of 17 LEM had a mean IAF of 9.87 (*SD* 0.91) and the 17 MEM had a mean IAF of 10.17 (*SD* 1.37). The difference between these subgroups is small and not significant (*t* = 0.76). The similarity of the mean IAF between both groups also indicates that the *resting* condition in meditators was appropriate for determining the individual frequency bands and likely not impaired by a possible inability of resting without meditating.

#### Resting state contrasts

**Figure 5:**
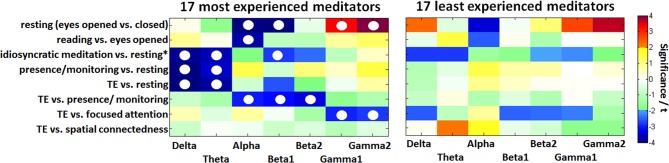
**Color-coded PSD differences shown as *t*-values resulting from the eight standard comparisons between psychophysiological states during the recording sessions for seven distinct frequency bands, averaged over the 17 most-experienced and the 17 least-experienced meditators**. The colors are coding *t*-values while all orange-red boxes are positively significant on a 5% level and all bluish values are negatively significant. White, yellow, and green boxes are non-significant. Fields marked with a white circle were significant after FDR adjustment on the 0.05 level.

Figure 5 illustrates the contrast between states for comparison of the two groups of the 17 MEM compared with the 17 individuals with the least experience. After FDR adjustment, none of the differences in the LEM, but several differences in the MEM group remained significant. In the experienced participants the contrast between *eyes open* and *eyes closed resting* (less alpha, more gamma activity) is much more pronounced, showing significant differences in alpha (*p* < 0.001), beta1 (*p* < 0.05), and gamma (*p* < 0.05). A similar, but less pronounced difference can be observed in the *reading* vs. *eyes open condition*: MEM show a significant decrease in alpha (*p* < 0.05).

#### Meditative state contrasts

Only the MEM show significant delta (*p* < 0.05) and theta (*p* < 0.05) power during *idiosyncratic meditation* compared to resting^*^ (Figure 5). The MEM also have decreased power in beta1 (*p* < 0.05).

Whereas for the MEM, there are significant differences between the *resting (eyes closed)* state and the distinct meditative conditions, this is not true for the LEM, who show no significant differences between *resting*, *presence/monitoring* and the state of *TE*.

Experienced meditators exhibit a stronger deactivation in the lower bands than non-experienced individuals, when instantiating the state of *TE*. We can see a more pronounced acitivity in the alpha to beta2 bands in the state of *presence/monitoring*, and a stronger activation in the gamma bands in *focused attention*. Only *TE* and *spatial connectedness* are almost identical states for the MEM, but distinct from all other meditative conditions or the *resting* state. This finding is in consonance with the one seen in the group of 30 experienced meditators.

#### Regression analysis (Q4b)

A regression analysis across all 50 participants using Spearman rank partial correlations of band power with meditation experience in hours, controlled for age, yielded *r*-values below 0.3 in most regions and frequencies; most of them were negative in all comparisons. Mainly in the delta band greater values were reached. Correction of *p*-values using the FDR adjustment eliminated almost all significant differences. Negative correlations larger −0.3 which remained significant could be observed in the *TE* vs. *focused attention* task comparison prodominantly in temporal, right parietal and occipital regions.

## Discussion

### Global PSD comparisons (Q1)

The first two comparisons present generally well known results. Opened eyes are associated with an alpha blocking, i.e., a decrease in alpha compared to closed eyes as well as an increase in gamma activities to be explained with increased visual and structural processing.

We have explicitly contrasted three meditative conditions to the normal *resting* state: the *idiosyncratic meditation*, the state of *presence/monitoring* and the task of *TE*. Even on the highest reduction level GFP comparisons reached significance in three of those comparisons. As the GFP could be strongly dominated by the delta band often showing high amplitudes we only discuss the spectrally resolved results. On that level of analysis our study showed that *resting* expressed higher delta and theta activity than any other meditative state. This contradicts the often reported theta increase during meditation in experienced meditators. However, theta increases were also associated with arousal and attention (O'Keefe, 1993; Klimesch, 1999), decreases in theta power have been found in motor tasks or imagined motor tasks, or more generally tasks requiring no input from the environment (Autret et al., 1985; Hansen et al., 1993; Weiss et al., 1995). Interpretations about the *TE* state that is characterized by less alpha and beta power in contrast to *presence/monitoring* and less gamma power in contrast to a *focused attention* task will be made in the discussion of spatially detailed activities [section Differences of Thoughtless Emptiness (TE) (Q2b)]. Additionally, it seems to be important to discuss possible artifacts which might lead to misinterpretations of our results.

### Considerations about artifacts

High frequency muscle artifacts would be expected to show up in the beta and gamma frequencies, mainly in areas closer to the face or neck. Also, these artifacts usually exceed the narrow band of neural activity and spread into higher frequencies. Facial muscle tensions especially show up in prefrontal, temporal and occipital areas but much less in frontal, central and parietal areas. In contrast, movements of head, eyes, etc. could produce an increase in delta power. As participants were sitting still, possible movement artifacts were limited to small time frames which do hardly influence our results through the calculation of the median.

When evaluating the results from the four meditative tasks (recording session 3) following the *idiosyncratic meditation* (recording session 2) we need to remember that there was only a short break of a few minutes after a 20–30 min *idiosyncratic meditation* and we cannot guarantee a return to a baseline activity during this break. Therefore, comparisons of the instructed meditative tasks from the third session with *resting* from the first recording session have to be handled with caution. However, the obvious differences between tasks also indicate that meditators were able to switch quickly between these mental states. Subjective reports also state that. This issue could have been avoided by introducing a final *resting* task at the end and compare this with the initial resting conditions or by introducing another *resting* task between recording sessions 2 and 3.

### Detailed PSD comparisons (Q2)

#### Differences to resting states (Q2a)

***States within baseline recording***. Decreased beta 1 during *resting with opened eyes* compared to *closed eyes* was predominantly found in highly experienced meditators related to frontal midline, mid-central, parietal, and occipital areas. High beta was also reduced in parietal and occipital areas. This is in line with Wróbel (1998) who found occipital beta to decrease in amplitude with increased visual attention. The increased frontal and temporal beta bands as well as global gamma increases during *opened eyes* reflect the higher cognitive load. This might also be responsible for further alpha blocking in the *reading* condition.

***Meditative states compared to resting***. All comparisons of *meditative states* with *resting* (*eyes closed* or resting^*^) showed almost globally significant decreases in delta and theta power which could also be observed in the not displayed comparisons to *focused attention* and *spatial connectedness*. This finding is in line with the results from Dunn et al. on mindfulness meditation (Dunn et al., 1999). Delta effects have not been reported in most studies on meditation, and it is unclear whether it has been systematically analyzed (Cahn et al., 2010). Delta occurs usually during slow wave sleep, and the restorative value of sleep is linked to the periods of delta waves when human growth hormone is secreted (Feinberg, 2000). Synaptic plasticity in the cortex is supported by delta waves as well (Steriade and Timofeev, 2003). LOC due to general anesthesia is also associated with a predominance of slow-frequency oscillations (Gugino et al., 2001; John et al., 2001). However, delta increases also during mental tasks (Dolce and Waldeier, 1974; Valentino et al., 1993; Fernández et al., 1995) and correlates with performance (Vogel et al., 1968). It has been suggested that delta activity is related to attention and internal processing (Harmony et al., 1996). Generally, decreases in slow frequencies and increases in fast frequencies characterize EEG profiles under influence of psychostimulants (Hermann, 1982), which usually enhance alertness, wakefulness, concentration or mood (Nehlig et al., 1992; Koelega, 1993; Seidl et al., 2000). A decrease of these frequencies in our participants might indicate that they were indeed fully conscious during the meditative states.

Considering these findings, the decreased low frequency activity during *TE, presence/monitoring* and *idiosyncratic meditation* might be related to increased calmness and possibly also enhanced mood and concentration associated with consistent meditation practice. This tallies nicely with recent findings that mindfulness and transcendental meditation (TM) practice improves ADHD symptomatology in children (Grosswald et al., 2008; Zylowska et al., 2008; Van der Oord et al., 2012). Interestingly, a study analyzing the EEG changes evoked by the smoking of a cigarette reported decreased delta and theta power and increased alpha power compared to a sham smoking condition (Knott, 1988; Knott et al., 1998). This correlates to the picture of *presence/monitoring* vs. *resting*. Theta and alpha rhythms have been proposed to reflect top-down information processing involving attention and working memory retention (Razumnikova, 2007). Consistently, the task *presence/monitoring* falls into the category of open monitoring meditation and is characterized by spatial awareness and attentiveness. It is supposed to reflect a reduction in episodic memorization, planning and problem solving. Our results confirm that the state of *presence/monitoring* can be clearly opposed to drowsiness, sleepiness, sleep and other non-conscious states as described in the introduction.

In contrast, *resting with eyes closed* can be regarded as a wakeful and alert state which is associated with so-called mind-wandering activities which might keep the sensorimotor system alert. We therefore will focus more on the default mode processes active in *resting* conditions. According to the introduction, the condition of *presence/monitoring* should then move the RSN toward the DMN, and probably an increased attentiveness. The contrast of *presence/monitoring* and *resting* nicely reflects the typical DMN activations by showing decreased theta and delta and increased alpha and gamma corresponding to the DMN according to Jann et al. (2010), the default mode RSN1 and the self-referential RSN6 but not the RSN2. The loss of memorization might be the reason for the theta decrease. Dunn et al. (1999) also reported an increase in alpha power as observed during the *presence/monitoring* condition. The increased right temporal gamma power is discussed in the section Thoughtless Emptiness and Idiosyncratic Meditation (Q3).

For all comparisons of meditative states with the resting state it should be noted that some meditators might automatically have been in a meditation state during the *resting* task even if they were instructed not to do so. This effect decreases the effects on one hand but on the other hand gives rise to the question how resting states are different in meditators and non-meditators.

#### Differences of thoughtless emptiness (TE) (Q2b)

***TE vs. resting (eyes closed)***. Similarly to the *presence/monitoring* task the theta and delta waves were globally and highly significantly reduced in intended *TE*. The pattern presents no alpha increase but a decrease in beta frequencies (predominantly frontal to occipital midline) as illustrated in Figure 4E. According to the discussion above this suggests that *TE* is neither an attentive state, nor is it related with sleep. Frontal beta power is decreased during *idiosyncratic meditation* and *TE*. Frontal beta synchronization is associated with stimulus assessment and decision making (Kropotov, 2008). A decrease might thus be related to an absence of these processes, indicating a non-judgmental attitude as aimed for during meditation. *TE* seems not to activate the DMN considering the highly significant decreases in alpha and beta waves.

***TE vs. presence/monitoring***. Desynchronizations in central areas in the alpha (or mu-rhythm band) and in the beta1 band have been associated with activation of the sensorimotor cortex and reported during preparation and execution of voluntary movement (Pfurtscheller, 1981; Pfurtscheller and Berghold, 1989; Toro et al., 1994; Pfurtscheller et al., 1996; Leocani et al., 1997) as well as during imagined movements (Pfurtscheller and Neuper, 1997; McFarland et al., 2000) and also attention to body parts (Jones et al., 2010). Alpha desynchronization has also been associated with improved information transfer through sensorimotor pathways (Klimesch, 1999; Pfurtscheller and Lopes da Silva, 1999). This suggests that the highest synchronization of the resting rhythms in motor and premotor areas observed in the *presence/monitoring* state (Figures 4D,F) suggest the largest quieting and possibly a decoupling of sensorimotor areas, movement-related areas, and parietal spatial processing areas from those processes still required for a wakeful consciousness. Those resting rhythms were not predominant in *TE*. However, the strongly decreased wide-spread beta frequencies also suggest a clearly reduced processing in areas attributed to motor-tasks, vision and cognition, since increasing beta2 activity is usually observed in cognitive tasks requiring greater attention (Razumnikova, 2007). Beta1 activity has been associated with the integration of different sensory qualities into a unified perception (Von Stein and Sarnthein, 2000; Hanslmayr et al., 2007), and has been proposed to be part of the experience of unity during meditation (Travis and Shear, 2010). The findings of significant beta1 decreases in *TE* suggest that *TE* does not involve such integration processes.

***TE vs. focused attention***. Lower gamma in *TE* can also be interpreted as an increase of gamma during *focused attention* which is in line with a recent study of mindfulness meditation (Cahn et al., 2010). It also has been hypothesized by Travis and Shear (2010) that *focused attention* during meditation might lead to higher beta2 and gamma activity. Our findings support this hypothesis at least for the gamma band by showing that *TE* contrasts with *focused attention* in a wide-spread deactivation of gamma and in the left temporal area with beta2. In the states of *presence/monitoring* and *focused attention*, posterior gamma activity was increased, which has also been reported during meditation of mindfulness practitioners. This is supposedly associated with changes in self-referential and attentional networks and may be related to momentary sensory awareness, or to conscious representation of mental content (Cahn et al., 2010; Berkovich-Ohana et al., 2012). In other words, these functions were possibly reduced in the state of *TE*.

***TE vs. spatial connectedness***. TE showed very similar brain activation to the spatial connectedness task, indicating a mental similarity between the sensations of nothingness and multidirectional infinite extension, thus a feeling of oneness.

***General finding***. Generally, the state of intended TE offered the lowest spectral activities across all frequency bands. One study using a clustering analysis of wavelet features identified five patterns involved in meditation, one of which they identified as particularly outstanding, showing a decrease in EEG power over 8 channels (F3, F4, C3, C4, P3, P4, O1, O2), and being associated with the subjective feeling of a unified, egoless and blessed state (Chang and Lo, 2005).

To summarize, *TE* constitutes a conscious and wakeful state of reduced resting rhythms, but also reduced higher frequencies suggesting diminished conscious representations of mental contents as well as reduced self-referential and attentional networks and maybe sensory awareness. Absence of activations related to decision making suggest a non-judgmental attitude. Physiologically, it represents itself similar to an instructed *spatial connectedness*.

### Thoughtless emptiness and idiosyncratic meditation (Q3)

The *idiosyncratic meditative* mental states of 30 long-term meditators were characterized by a decrease of power in the slow frequency bands and partially also in the faster frequencies. This result is similar to the reported decrease in power over all frequency bands during a state of “sacred, unified, egoless, and blessed meditation” (Chang and Lo, 2005), and also consistent with an early suggestion, that during advanced stages of meditation, cognitive functions are automatized or inhibited, leading to a reduction in cortical activity (Earle, 1981). Looking at the contents and strategies the meditators reported for their idiosyncratic meditation style 10/30 mentioned a kind of TE as aim. Another 10/30 used mindfulness techniques, 4/10 visualizations, and again 10/30 reported of the experience of connectedness or unity. Probably, the similarity between the *idiosyncratic meditations* with *TE* and the *spatial connectedness* can be explained by the fact that 20/30 meditators had similar meditation habits which physiologically differed from a state of *presence/monitoring*.

Overall, the brain activity during *idiosyncratic meditation* was similar to that during the intended *TE* condition. A remarkable difference is the reduction of alpha power during *idiosyncratic meditation* in the prefrontal, central and mid-parietal regions. This might be due to attention directed differently toward the body or the environment. The central alpha desynchronization during *idiosyncratic meditation* suggests that the *idiosyncratic meditations* are stronger loaded with imagery.

A specific characteristic of *TE* and the *idiosyncratic meditations* seems to be the right temporal gamma increase compared to the resting state. The right anterior temporal lobe has been associated with the solution of insight problems, with gamma frequencies indicating the event of the insight (Jung-Beeman et al., 2004). Maybe during meditation the meditators reached a state comparable to prolonged insight. In *TE*, the right temporal gamma effect is smaller and as we do not know about thoughtless insights, we cannot explain this effect in the *TE*. Similarly, a decrease of beta power in the parieto-occipital and centro-temporal regions was found during the solution of insight problems (Sheth et al., 2009). This would probably support the findings in *idiosyncratic meditation* but not in the *TE* task.

### Proficiency of meditators (Q4)

Meditation experience seemed to have a noticeable influence on brain activity. The observed contrasts we discussed above were much more prominent in the 17 highly experienced meditators than in the 17 least experienced ones. The less experienced meditators showed no significant differences between the states of *presence/monitoring* or *TE* and the *resting* condition, indicating that they could not reach or maintain the desired mental state and still might have been cognitively active. The great similarity between *resting* state, *presence/monitoring* and *TE* in the LEM may indicate that they were generally less able to distinguish or maintain different mental states. Differences between baseline conditions are also considerably more pronounced in MEM, further supporting the theory of their enhanced ability to switch between mental states. Also taking into account that none of the differences in the LEM were significant after FDR adjustment, practicing meditation seems to be a tool for enhancing the ability to modulate brain activities and thereby to enable oneself to enter a larger variety of states of consciousness. The observation that the long idiosyncratic meditation corresponds to similar albeit not significant EEG activity in the LEM as in the MEM, might indicate that some LEM were able to reach the desired state or maintain it at least some of the time.

These proficiency related differences did not become visible in a regression analysis between meditation experience and PSD data. While the correlation between meditation experience and the significantly observed differences between states was only weak, it becomes visible in a two-group comparison according to Figure 5.

Our results do not support the findings by previous studies that associated increased meditation experience with increased theta power (Kasamatsu and Hirai, 1966; Wallace, 1970; Wallace et al., 1971; Corby et al., 1978), or an increase of theta power and a decrease of alpha power (Pagano and Warrenburg, 1983; Jacobs and Lubar, 1989; Milz et al., 2011). Inanaga (1998) suggested a negative correlation between frontal midline theta and anxious or frustrated experience which might arise due to inability of subjects to reach or maintain the desired states; this is also in contradiction to our data, which suggests decreased theta power in most brain areas in the MEM but no changes in the LEM compared to the *resting* state.

Treatment of Alzheimer's patients with acetylcholinesterase inhibitor to enhance cholinergic neurotransmission resulted in globally decreased theta power (Brassen and Adler, 2003). The reduced theta power in our MEM during *idiosyncratic meditation*, *presence/monitoring*, and *TE* might, speculatively, be taken in support of the finding that long-term meditators show increased cortical thickness in the putamen and no cognitive decline compared to age matched controls (Pagnoni and Cekic, 2007). The putamen is part of the basal ganglia. Acetylcholinergic activity is mainly derived from the nucleus basalis Meynert and its failure to provide adequate cholinergic activation is taken to be one of the starting points of Alzheimer's dementia (Hasselmo and McGaughy, 2004). It might well be the case that what we see in our data is a heightened efficiency of attentional networks that are vital for all other cognitive processes.

Data associating meditation experience with increased theta-power is thus not reliable to identify the neural correlates underlying the state of *TE*. These results confirm that research on distinct mental states should focus on analysis of experienced meditators, as we did in the previous sections of the results. The activity patterns observed in the larger group of 30 experienced meditators are very similar to those from the 17 MEM and the larger sample provides for greater generalizability and validity of the results.

In summary, our data suggest that proficient meditators are more efficient in recruiting cognitive resources while at the same time staying highly conscious and apparently in a generally more pleasant affect. They seem to be able to switch more effectively and more clearly between different states of consciousness and to also hold a state of pure consciousness in a highly awake yet contentless state.

## Conclusion

The results demonstrate that during *idiosyncratic* forms of *meditation* (mainly Zen-Buddhism, Sahaja Yoga, and Western contemplative methods) most EEG band activities were down-regulated compared to a normal resting awake state. A very similar picture could be seen for experienced meditators who were asked to enter a state of *TE*.

The missing increase of slow wave activity (theta and delta waves) during *TE* could serve as an important indicator that wakeful consciousness is maintained while, at the same time, giving up most of the information processing activities. It is worthwhile pointing out that an implicit teaching of cognitive science suggests that consciousness without content is not possible, dating back to Franz Brentano's notion of intentional consciousness. This states that consciousness is always accompanied by some intention toward which it is directed (Brentano, 1982, p. 21). Our data suggest that this might not be the case: even in a state of reduced cognitive processing clear consciousness seems to be possible in experienced meditators. Their phenomenological first person accounts can also be supported by their neurophysiological data.

These results suggest that states of TE are different from higher cognitive and mentally focused states and from default-mode resting states as well as states of sleep and drowsiness; this can be observed in a general down-regulation of brain activities that also affect the resting rhythms and the lower brain frequencies such as alpha, theta and delta. These findings contribute to solving the question of which brain processes may be required for the experience of being conscious with and without content.

### Conflict of interest statement

The authors declare that the research was conducted in the absence of any commercial or financial relationships that could be construed as a potential conflict of interest.

## Footnotes

^1^$d=\frac{x_{1}-x_{2}}{\sqrt{(s_{1}^{2}+s_{2}^{2})/2}}$ with *x*_1_ and *x*_2_ being the mean PSD values and *s*_1_ and *s*_2_ the estimated variances, according to Cohen (1988).
